# Changes in Reactive Oxygen Species, Antioxidants and Carbohydrate Metabolism in Relation to Dormancy Transition and Bud Break in Apple (*Malus* × *domestica* Borkh) Cultivars

**DOI:** 10.3390/antiox10101549

**Published:** 2021-09-29

**Authors:** Sangeeta Sapkota, Jianyang Liu, Md Tabibul Islam, Sherif M. Sherif

**Affiliations:** Alson H. Smith Jr. Agricultural Research and Extension Center, School of Plant and Environmental Sciences, Virginia Tech, 595 Laurel Grove Road, Winchester, VA 22602, USA; sangee7@vt.edu (S.S.); liujy4@vt.edu (J.L.); tabibul@vt.edu (M.T.I.)

**Keywords:** bud dormancy, spring frost, bloom delay, pome fruits, apple, ROS, antioxidants

## Abstract

Understanding the biochemical mechanisms underlying bud dormancy and bloom time regulation in deciduous woody perennials is critical for devising effective strategies to protect these species from spring frost damage. This study investigated the accumulation profiles of carbohydrates, ROS and antioxidants during dormancy in ‘Cripps Pink’ and ‘Honeycrisp’, two apple cultivars representing the early and late bloom cultivars, respectively. Our data showed that starch levels generally declined during dormancy, whereas soluble sugars increased. However, the present study did not record significant alternations in the carbohydrate accumulation profiles between the two cultivars that could account for the differences in their bloom dates. On the other hand, H_2_O_2_ accumulation patterns revealed an apparent correlation with the dormancy stage and bloom dates in both cultivars; peaking early in the early-blooming cultivar, sustaining high levels for a longer time in the late-blooming cultivars, and fading by the time of bud burst in both cultivars. Also, the redox balance during dormancy appeared to be maintained mainly by catalase and, to a lesser extent, by glutathione (GSH). Overall, the present study concludes that differences in ROS and the bud redox balance could, at least partially, explain the differences in dormancy duration and bloom date among apple cultivars.

## 1. Introduction

Temperate woody perennials annually cycle between growth cessation and growth resumption phases in synchrony with seasonal changes in temperature and photoperiod. Bud dormancy is critical for the tree to survive under potential freezing and subfreezing temperatures in winter. Bud dormancy can be triggered either by low temperatures and short days, or by low temperatures only, such as in apples and pears [1]. The dormancy stage is generally categorized into endodormancy, in which growth cessation is largely due to internal signals within the bud; and ecodormancy, in which growth inhibition is due to external environmental conditions [2]. Buds require a certain number of chill units, referred to as chilling requirements (CR), to release from endodormancy, and a certain number of heat units, referred to as heat requirements (HR), to release from ecodormancy. The fulfillment CR and HR is interrelated and crucial for homogenous budburst and flowering in spring. After dormancy release, buds lose their cold hardiness and become more sensitive to spring freezes, which are predicted to increase in the future due to global climate change [3]. Bloom delay using plant growth regulators (PGRs) and vegetable oil-based adjuvants has been suggested as an effective frost avoidance strategy, especially in grapes and stone fruits [4,5,6]. However, in apples and other pome fruits, such strategies are lacking, probably due to the poor understanding of dormancy and flowering time regulation mechanisms in these crops. Hormonal regulation has been reported to play a significant role in dormancy; however, limited studies have focused on unravelling the potential role of carbohydrates, reactive oxygen species (ROS), and antioxidants during the dormancy-regrowth cycle, especially in pome fruits.

Dormancy release and bud burst involve numerous physiological and biochemical changes that include, but are not limited to, changes in hormone hemostasis, lipid mobilization, carbohydrate interconversion and transport, and redox processes [7,8,9,10]. In addition to being the primary source of carbon and energy, carbohydrates also play an important role in abiotic stress tolerance [11]. Indeed, the role of carbohydrate in osmoregulation and cryoprotection is substantial for bud survival under subfreezing temperatures in winter. In essence, the accumulation of soluble sugars as a result of starch degradation during dormancy is believed to reduce the free water at the cellular levels, increasing the freezing tolerance of dormant buds [12]. During endodormancy, a decrease in the starch concentration and increase in the total soluble sugar (TSS) was observed in pear [13,14], apple [15,16], walnut [17,18], grape [19], and poplar [20]. Sugars also provide the carbon skeleton for metabolites necessary for bud differentiation, growth and development. Changes in sugar levels during dormancy and dormancy release have been demonstrated in several woody perennials including apple [21], apricot [22], grape [23], Japanese pear [24], peach [25], and sweet cherry [26].

The exposure of plants to stressful conditions promotes the production of ROS, such as hydrogen peroxide (H_2_O_2_) and superoxide (O_2_^•−^), which are key components in the redox signaling pathways when present below the lethal levels [27,28]. Redox reactions and turnover of (ROS) in cells can be carried out through non-enzymatic and enzymatic antioxidants that maintain ROS at a sub-lethal level. Endodormancy release and bud burst is usually accompanied by increased levels of H_2_O_2_ and O_2_^•−^ in the buds [29]. In Japanese pear, sufficient chilling exposure increases H_2_O_2_ content of flower buds with the onset of endodormancy release, whereas insufficient chilling exposure fails to change H_2_O_2_ content [30]. Also, treating buds with hydrogen cyanamide (HC), a bud break agent, causes a transitory up-regulation of H_2_O_2_ leading to dormancy release in grapes [31,32]. O_2_^•−^ is generated by plasma membrane-based NADPH oxidase that transfers electrons from cytoplasmic NADPH to oxygen. NADPH oxidase has been reported to promote dormancy release in Arabidopsis seeds and potato tubers [33,34]. Indeed, treating with NADPH oxidase inhibitors suppresses seed germination in barley [35] and tuber sprouting in potato [33]. However, little is known about the role of NADPH oxidase during dormancy transition in deciduous fruit trees. Both non-enzymatic antioxidants such as glutathione and enzymatic antioxidants such as superoxide dismutase, catalase, glutathione peroxidase and glutathione reductase play crucial roles in maintaining optimum levels of ROS during dormancy [25,36,37,38,39,40]. Comparisons of several transcriptomic studies showed induction of the same set of genes after exposure to chilling, HC or H_2_O_2_. These genes include superoxide dismutase, catalase, glutathione-s-transferase, glutathione reductase, ascorbate peroxidase as well as redox-related genes [29,41,42].

ROS’s role in regulating dormancy and bud burst is also tightly linked to the relative abundance of carbohydrates and phytohormones [43]. For instance, Liu et al. (2010) demonstrated that the rise of H_2_O_2_ in Arabidopsis seeds promotes dormancy release through stimulation of abscisic acid (ABA) catabolism and gibberellin (GA) biosynthesis [44]. Similarly, in barley seeds, NADPH oxidase induces ABA catabolism and α-amylase activity, which are prerequisite for seed germination [35]. Along the same vein, exogenous application of HC which is widely reported to induce H_2_O_2_ production, decreases endogenous ABA levels in grape [45,46] and sweet cherry [47] by promoting ABA degradation and inhibiting ABA synthesis. In walnut, redox interaction rather than sugar metabolism was found to govern bud dormancy release [18]. Indeed, enzymes and proteins that facilitate oxygen reduction to generate H_2_O_2_, O_2_^•−^ and hydroxyl (^•^OH) radicals can play central roles in signal transduction pathways by changing the oxidation and reduction of proteins cysteinyl thiols, and subsequently altering protein functions and characteristics [48]. These studies and others signify the importance of ROS and its potential interactions with different pathways throughout the dormancy-regrowth cycle. However, how sugars and ROS interact during bud dormancy and how their accumulation kinetics correspond to CR, HR and bloom time is largely unclear, especially in apples.

The aim of this study was to investigate the accumulation patterns of sugars, ROS and antioxidants in relation to bud dormancy progression and flowering time in apple (*Malus* × *domestica*). To this end, we used two apple cultivars with contrasting bloom dates, ‘Cripps Pink’ and ‘Honeycrisp’, and collected flower buds from these cultivars at different times of endodormancy and ecodormancy to quantify the levels of sugars and ROS related compounds and enzymes. These two cultivars were previously used by Sapkota et al., 2021, to monitor the levels of major plant hormones (e.g., ABA, cytokinins, GA and jasmonic acid) throughout the dormancy cycle. Given the anticipated interactions among these plant hormones, ROS and carbohydrate metabolism, the present investigation can deepen our understanding of the regulation of dormancy and flowering in apple and lay the foundation for effective frost mitigation strategies.

## 2. Materials and Methods

### 2.1. Plant Materials & Samples Preparation

The detailed description of the field experiment was presented in our previous study [49]. Briefly, in 2019, two apple cultivars, ‘Honeycrisp’/B.9 and ‘Cripps Pink’/M.9, at the Alson H. Smith Jr. Agricultural Research and Extension Center (AREC) in Winchester, VA, the United States (39.11, −78.28) were used. It is worth noting that B.9 and M.9 rootstocks have similar vigor and previous reports showed no significant differences between these rootstocks in stress tolerance [50] and bud break [51]. Three trees from each cultivar were randomly selected, each considered as a biological replicate. Spur buds were collected from each replicate at regular intervals of chilling hours (CH) (i.e., 200, 400, 600, 800, 1000 CH) and growing degree hours (GDH) (i.e., 1000, 2000, 3000, and 4000 GDH), respectively. At each sampling point, about 20 spur buds from each tree were collected, immediately frozen in liquid nitrogen, then stored at −80 °C until further use. Bud samples were homogenized in liquid nitrogen using a Geno Grinder (SPEX SamplePrep, Metuchen, NJ, USA). Approximately 100 mg of finely ground tissue was used from each sample for the quantification of soluble sugars, starch, ROS and antioxidants.

### 2.2. Estimation of Chilling and Heat Accumulation during Dormancy

**Figure 1:**
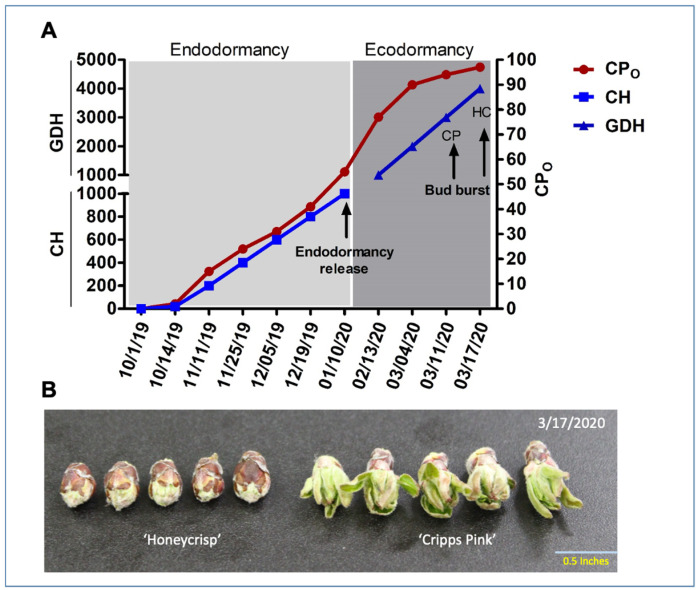
Accumulation of chilling hours (CH), chilling portions (CP_O_), and growing degree hours (GDH) in ‘Honeycrisp’ (HC) and ‘Cripps Pink’ (CP). Accumulation of CH, GDH, CPO, and major events are shown with arrows (**A**). An image showing HC and CP flower buds collected on the same day (**B**). The CH accumulation started on October 2019 reaching 20 CH (2 CPO) on 10/14/2019.

In this study, chilling and heat accumulation was estimated using CH and GDH models, respectively. A data logger (EasyLog, Lascar Electronics, Erie, PA, USA) was used to record the field temperatures at 10-min intervals (Figure 1A). CH were computed as the number of hours with average temperatures in the range of 0 and 7.2 °C [52]. GDH were calculated as follows: GDH = 0 (T ≤ 4.5 °C); GDH = T − 4.5 (4.5 °C < T ≤ 25 °C); GDH = 21.5 (T > 25 °C) [53]. The GDH were adjusted by excluding the GDH accumulated before the endodormancy release respective to each cultivar. The endodormancy induction and release time was evaluated by keeping branch cuttings from each cultivar in forcing conditions previously described by Sapkota et al., 2021 [49]. The ecodormancy release date was marked at the bud green-tip stage.

### 2.3. Carbohydrate Analysis

Carbohydrate analysis was performed according to Edwards et al. (2011) [54]. Briefly, soluble sugars were extracted from 100 mg of the bud tissue using 1 mL of 80% ethanol, followed by vortexing and centrifugation at 14,000 rpm (21,074× *g*) for 10 min. The supernatant was collected and this extraction step was repeated twice. Soluble sugars (sucrose, glucose and fructose) were determined using commercial enzyme assays (Megazyme, Bray Business Park, Bray, Co., Wicklow, Ireland). To determinate the starch levels, the pellet was dried overnight to remove ethanol completely. The dry pellet was suspended in 500 µL of deionized water and the solution was incubated at 80 °C for 20 min. The solution was then cooled and mixed with 400 µL of 200 mM acetate buffer (pH 5.1). A 100 µL of 2 U of amyl glucosidase and 40 U of α-amylase was added to the solution, followed by incubation at 50 °C for 24 h and centrifugation at 14,000 rpm (21,074× *g*) for 10 min. The glucose content in the supernatant was measured and starch content was estimated as glucose times 0.9.

### 2.4. Hydrogen Peroxide and Superoxide Quantification

The samples for hydrogen peroxide (H_2_O_2_) and superoxide (O_2_^•−^) were prepared according to Lei et al. (2006). In brief, 100 mg ground sample was mixed with 1.0 mL of 50 mM K-PO_4_ (pH 7) and incubated on ice for 10 min. The solution was then centrifuged at 12,000 rpm (15,483× *g*) at 4 °C for 10 min. The supernatant was used for H_2_O_2_ and O_2_^•−^ content evaluation.

For H_2_O_2_ assay, an aliquot of supernatant (200 µL) was mixed with 200 µL of 0.1% TiCl_4_ in 20% H_2_SO_4_ (*v/v*) and incubated for 5 min. The solution was centrifuged at 10,000 rpm (10,752× *g*) for 5 min at room temperature and the absorbance was recorded at 410 nm using a microplate reader (Synergy H1, Biotek, Winooski, VT, USA). The H_2_O_2_ content in the tissue was calculated using the extinction coefficient (e = 0.28 µ mol^−1^ cm^−1^).

For O_2_^•−^ analysis, an aliquot of supernatant (100 µL) was mixed with 25 µL of 10 mM NH_2_OH-HCl and incubated at 25 °C for 1 h. After incubation, 50 µL of 7 mM sulfanilamide and 50 µL of 7 mM α-naphthylamine were added to the solution and incubated at 25 °C for 20 min. The absorbance was recorded at 530 nm spectrophotometrically. The O_2_^•−^ content was calculated based on a standard curve prepared by serial dilutions of NaNO_2_ (2–250 ppm).

### 2.5. Extraction and Quantification of Glutathione

For the extraction of glutathione, 500 mg of ground bud tissue was mixed with 0.6 mL 5% sulfosalicylic acid, and centrifuged at 12,000 rpm (15,483× *g*) at 4 °C for 10 min. The content of reduced glutathione (GSH) and glutathione disulfide (GSSG) was determined using a GSH: GSSG kit following the manufacturer’s protocol (Oxford Biomedical Research Inc., Rochester Hills, MI, USA). The ratio of GSH: GSSG was calculated by simple division of the concentrations of GSH and GSSG.

### 2.6. Enzymatic Assays

100 mg ground bud tissue was extracted with 1 mL of cold potassium phosphate buffer (50 mM, pH 7). After centrifugation at 4 °C, the supernatant was directly used for protein content measurement and enzyme activity assays. Total soluble proteins were quantified according to the Bradford assay [55] and using the bovine serum albumin (BSA) for making a standard curve. The NADPH oxidase activity was determined spectrophotometrically at 492 nm as described by Kaundal et al. (2012) [56]. NADPH oxidase activity was determined based on its ability to generate O_2_^•−^ by the reduction of tetrazolium salt XTT. The activity of the catalase (CAT), superoxide dismutase(SOD), glutathione peroxidase (GPX), and glutathione reductase (GR) enzymes were determined spectrophotometrically using the colorimetric kits (BioVision Inc, Milpitas, CA, USA) and following the manufacturer’s instructions.

### 2.7. Statistical Analysis

ROS, sugar quantification and enzymatic activities data were analyzed using R statistical programming language (version 3.6.3). Student’s *t*-test was used to compare the means between the cultivars at each sampling time point. All values were expressed as mean ± SEM. Probabilities of *p* < 0.05 were considered statistically significant. Person’s correlation table was developed using metaboanalyst version 5.0 (https://www.metaboanalyst.ca/, accessed on 12 February 2021) at *p* < 0.05. The data of plant hormones (abscisic acid, ABA; gibberellin, GA_20_; cytokinin, trans-zeatin tZ; and jasmonic acid-Isoleucine, JA-Ile) to build the correlation matrix was retrieved from our already published article [49].

## 3. Results

### 3.1. Phenological Variations between ‘Honeycrisp’ and ‘Cripps Pink’

The phenological events in this study were recorded according to CH and GDH calculated for ‘Honeycrisp’ and ‘Cripps Pink’ during the 2019 and 2020 dormancy-regrowth cycle as detailed in our previous investigation [49]. In brief, the endodormancy release was achieved at 1000 CH (10 January 2020) in both cultivars; whereas, ecodormancy release occurred at 3000 GDH (11 March 2020) in ‘Cripps Pink’, and 4000 GDH (17 March 2020) in ‘Honeycrisp’ (Figure 1A,B).

### 3.2. Changes in Carbohydrate Levels during Endodormancy and Ecodormancy

**Figure 2:**
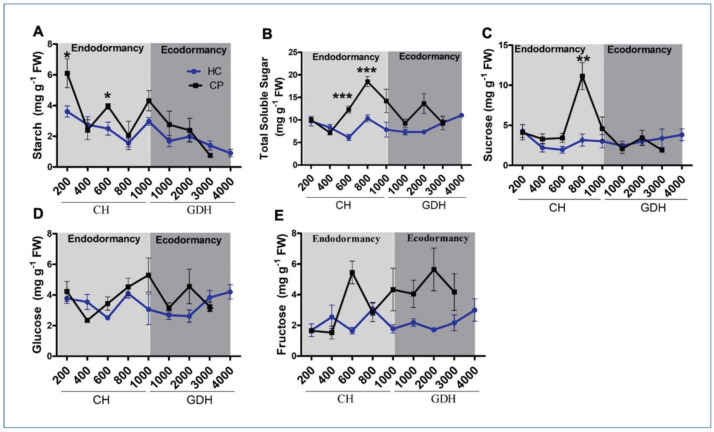
Starch (**A**), total soluble sugar (**B**), sucrose (**C**), glucose (**D**) and fructose (**E**) levels in two apple cultivars ‘Honeycrisp’ (HC) and ‘Cripps Pink’ (CP). Bud samples were collected from endodormancy induction to the bud break stage. The 200, 400, 600, 800, 1000 CH and 1000, 2000, 3000, and 4000 GDH corresponds to 11/11, 11/25, 12/05, 12/19, 1/10, 2/13, 03/04, 03/11, 03/17 dates in 2019–2020 respectively. Each point represents mean ± SEM of three biological replicates; *p*-value indicates comparison between ‘Honeycrisp’ and ‘Cripps Pink’ (* *p* < 0.05, ** *p* < 0.01, *** *p* < 0.001) using students *t*-test.

The starch content in the floral buds declined in both cultivars over the entire dormancy period, with some noticeable fluctuations (Figure 2A). At the beginning of endodormancy, the starch content in ‘Cripps Pink’ was 6.1 mg g^−1^, approximately 2.3 mg g^−1^ higher than ‘Honeycrisp’. The starch content in ‘Cripps Pink’ showed two remarkable rises at 600 and 1000 CH, whereas, ‘Honeycrisp’ showed only one rise at the time of endodormancy release (1000 CH). The starch content of the two cultivars both reached 0.5 mg g^−1^ at their respective budburst time of 3000 and 4000 GDH. The total soluble sugars (TSS) in ‘Cripps Pink’ showed a remarkable peak in the middle of endodormancy, while the TSS in ‘Honeycrisp’ remained relatively stable throughout both endodormancy and ecodormancy (Figure 2B). The TSS in ‘Cripps Pink’ increased from 10 mg g^−1^ at 200 CH, reaching its highest point of 20 mg g^−1^ at 800 CH, then decreased to 10 mg g^−1^ again at 1000 GDH, followed by a moderate rise at 2000 GDH before returning to 10 mg g^−1^ at budburst (3000 GDH). In contrast, the TSS in ‘Honeycrisp’ only showed some small changes during the dormancy, reaching a lowest point at 600 CH, followed by a slight rise at 800 CH, which was approximately half of ‘Cripps Pink’ at the respective time point.

To gain insights into the relative contributions of sugar forms to the TSS dynamics, we examined the concentrations of sucrose, glucose and fructose separately. In ‘Honeycrisp’, similar dynamic patterns were found in the three sugar forms, with glucose and fructose both peaking at 800 CH, corresponding with the peak of TSS (Figure 2C–E). In ‘Cripps Pink’, only the sucrose dynamics showed similarity to the TSS, with a prominent peak at 800 CH, 4 times higher than ‘Honeycrisp’ (Figure 2C). The glucose content peaked at 1000 CH and fructose peaked at 600 CH and 2000 GDH, which all contributed to the high TSS content at these timepoints (Figure 2D,E).

### 3.3. Accumulation Patterns of Hydrogen Peroxide, Superoxide Radicals and NADPH Oxidase during Dormancy

**Figure 3:**
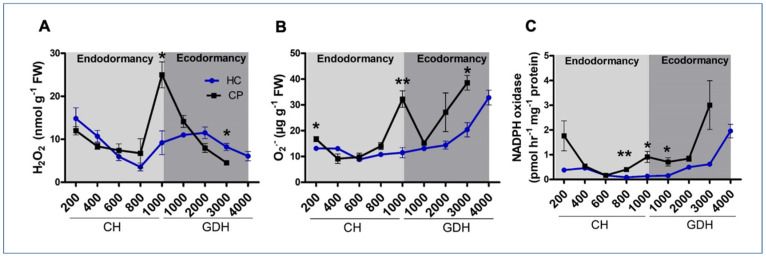
Profiles of hydrogen peroxide, H_2_O_2_ (**A**), superoxide radical, O_2_^•−^ (**B**) contents, and NADPH oxidase activity (**C**) in two apple cultivars ‘Honeycrisp’ and ‘Cripps Pink’ from the time of endodormancy induction to the bud break stage. Each point represents mean ± SEM of three biological replicates; *p*-value indicates comparison between ‘Honeycrisp’ and ‘Cripps Pink’ (* *p* < 0.05, ** *p* < 0.01) using students *t*-test.

At endodormancy induction, hydrogen peroxide (H_2_O_2_) content was high and started to decrease with the progression of endodormancy in both cultivars (Figure 3A). However, at endodormancy release (1000 CH), H_2_O_2_ level reached its peak and was 3 times higher in ‘Cripps Pink’ than ‘Honeycrisp’. The H_2_O_2_ level started to decrease with the progression of ecodormancy and reached its lowest point (~3 nmol g^−1^) at budburst in both cultivars. However, the decrease in H_2_O_2_ during ecodormancy started early (1000 CH) in ‘Cripps Pink’ compared to 2000 GDH for ‘Honeycrisp’.

The level of superoxide (O_2_^•^^−^) in both cultivars showed a general increase from the beginning of endodormancy to budburst (Figure 3B). In ‘Honeycrisp’, the O_2_^•^^−^ level remained between 10 and 12 µg g^−1^ during endodormancy, and increased gradually during ecodormancy, reaching its highest level of 30 µg g^−1^ at budburst. In ‘Cripps Pink’, O_2_^•^^−^ showed no significant changes at the early stages of endodormancy (400−800 CH), followed by a dramatic increase at 1000 CH and a dramatic decrease at 1000 GDH. The levels of O_2_^•^^−^ increased again with the progression of ecodormancy reaching its peak of 40 µg g^−1^ at budburst.

The NADPH oxidase activity during dormancy followed a similar pattern in the two apple cultivars, with both showing low NADPH oxidase activity during the endodormancy stage and a significant increase at their respective time of budburst (Figure 3C). It was noticeable though that NADPH oxidase activity in ‘Cripps Pink’ was higher than ‘Honeycrisp’ from 800 CH to 1000 GDH. At budburst, the NADPH oxidase activity in ‘Cripps Pink’ reached 3 pmol hr^−1^ mg^−1^; 1.5 times higher than ‘Honeycrisp’, but that difference was not statistically significant.

### 3.4. Redox Balance through Non-Enzymatic Antioxidants

**Figure 4:**
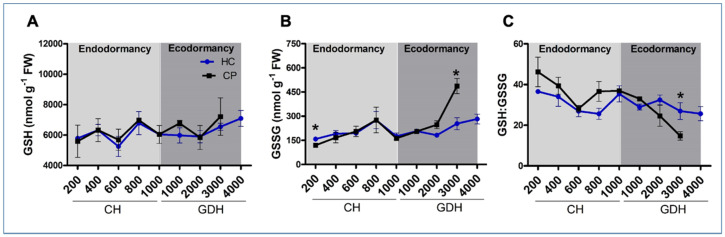
Changes in reduced glutathione, GSH (**A**), glutathione disulfide, GSSG (**B**) contents, and reduced to oxidized glutathione, GSH: GSSG (**C**) in two apple cultivars, ‘Honeycrisp’ (HC) and ‘Cripps Pink’ (CP) from the time of endodormancy induction to the bud break stage. Each point represents mean ± SEM of three biological replicates; *p*-value indicates comparison between ‘Honeycrisp’ and ‘Cripps Pink’ (* *p* < 0.05) using students *t*-test.

The reduced GSH content showed slight, yet insignificant, increase during endodormancy and decreased at endodormancy release (Figure 4A). During ecodormancy, the increase in reduced GSH started earlier in ‘Cripps Pink’ compared to ‘Honeycrisp’. The disulfide glutathione (GSSG) content was 25 times lower than reduced GSH throughout dormancy in both the cultivars. The GSSG content increased with the progression of endodormancy but decreased at endodormancy release followed by an increase during ecodormancy (Figure 4B). The GSSG in ‘Cripps Pink’ increased rapidly during ecodormancy and reached its peak at bud burst. A similar pattern of increase was observed in ‘Honeycrisp’, which reached its peak at bud burst, but was 2 times lower than ‘Cripps Pink’. The GSH: GSSG ratio decreased with the progression of endodormancy in both the cultivars (Figure 4C). The GSH: GSSG ratio increased toward the onset of endodormancy release and the increase started (800 CH) in ‘Cripps Pink’, 200 CH earlier than ‘Honeycrisp’. After endodormancy release, the GSH: GSSG ratio continuously decreased reaching its minimum at bud burst in both the cultivars. The ratio was 3 times lower in ‘Cripps Pink’ due to rapid decline during ecodormancy compared to ‘Honeycrisp’.

### 3.5. Redox Balance through Enzymatic Antioxidants

**Figure 5:**
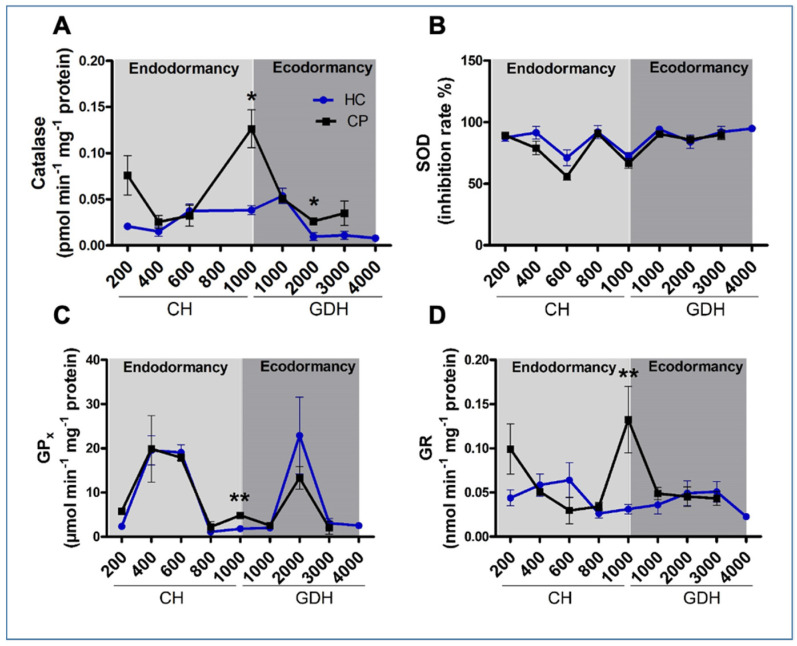
The enzymatic activities of catalase (**A**), superoxide dismutase, SOD (**B**), glutathione peroxidase, GPx (**C**) and glutathione reductase, GR (**D**) in two apple cultivars ‘Honeycrisp’ and ‘Cripps Pink’. Bud samples were collected from endodormancy induction to the bud break stage. Each point represents mean ± SEM of three biological replicates; *p*-value indicates comparison between ‘Honeycrisp’ and ‘Cripps Pink’ (* *p* < 0.05, ** *p* < 0.01,) using students *t*-test.

In general, the catalase activities in both cultivars were higher during the period near the endodormancy release than the early stage of endodormancy and later stage of ecodormancy (Figure 5A). At 200 CH, the catalase activity in ‘Cripps Pink’ was 0.075 pmol min^−1^ mg^−1^ and decreased quickly to 0.03 at 400 CH, then assuming a remarkable increase and reaching 0.125 pmol min^−1^ mg^−1^ at 1000 CH. The catalase activity in ‘Cripps Pink’ decreased rapidly during ecodormancy and remained low until budburst. In contrast, the catalase activity in ‘Honeycrisp’ increased from 0.02 pmol min^−1^ mg^−1^ at 200 CH to 0.03 pmol min^−1^ mg^−1^ at 600 CH, followed by another slight increase until 1000 GDH, before it decreased to 0.01 pmol min^−1^ mg^−1^ at 2000 GDH and remained unchanged until budburst at 4000 GDH. It can be noticed that catalase activity in ‘Cripps Pink’ was 2.5 times higher than ‘Honeycrisp’ at 1000 CH and 2000 GDH.

The overall superoxide dismutase (SOD) activities were highly similar between the two cultivars, especially during the ecodormancy period (Figure 5B). The inhibition rates of SOD were both 90% at the beginning of endodormancy, and decreased to 75 and 55% for ‘Honeycrisp’ and ‘Cripps Pink’, respectively, at 600 CH. After rising back to the initial rate of 90% at 800 CH, the SOD activities in both cultivars remained at this level until budburst, except for a noticeable drop at 1000 CH. There was no significant difference between the two cultivars at any timepoint.

Dramatic fluctuations of the glutathione peroxidase (GPx) activities were observed in both cultivars throughout the dormancy period (Figure 5C). The GPx activities were 2.0 and 5.0 µmol min^−1^ mg^−1^ for ‘Honeycrisp’ and ‘Cripps Pink’, and both increased rapidly to 20.0 µmol min^−1^ mg^−1^ at 400 CH, plateauing until 600 CH, at which both declined sharply to 2.0 µmol min^−1^ mg^−1^ at 800 CH. After remaining at the low level until 1000 GDH, the GPx activities of ‘Cripps Pink’ (12 µmol min^−1^ mg^−1^) and ‘Honeycrisp’ (22 µmol min^−1^ mg^−1^) both reached a peak at 2000 GDH, followed by a rapid decrease to 2.0 µmol min^−1^ mg^−1^, which remained until budburst. It is noticeable that the GPx activity in ‘Cripps Pink’ was higher than in ‘Honeycrisp’ at 1000 CH, corresponding to the time of endodormancy release.

The glutathione reductase (GR) activities showed different trends in the two cultivars during endodormancy and ecodormancy (Figure 5D). At early stage of endodormancy, the GR activities in the two cultivars followed opposite trends; ‘Cripps Pink’ decreasing from 0.1 nmol min^−1^ mg^−1^ at 200 CH to 0.03 nmol min^−1^ mg^−1^ at 600 CH, while ‘Honeycrisp’ increasing from 0.05 to 0.07 nmol min^−1^ mg^−1^ during the same period. After reaching 0.3 nmol min^−1^ mg^−1^ at 800 CH, the GR activity in ‘Cripps Pink’ peaked (1.3 nmol min^−1^ mg^−1^) at 1000 CH, 3 times higher than ‘Honeycrisp’, then declined to 0.5 nmol min^−1^ mg^−1^ at 1000 GDH, remaining at this level until budburst. In contrast, the GR activity in ‘Honeycrisp’ increased steadily since 800 CH until 3000 GDH, and declined slightly at budburst (4000 GDH).

### 3.6. Persons Correlations between Hormones, Sugars and ROS

**Figure 6:**
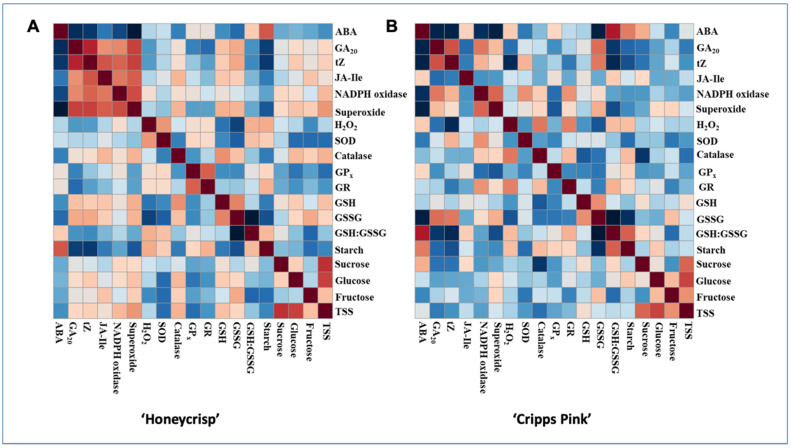
Heatmap analysis of Pearson’s correlation among variables measured in two apple cultivars, ‘Honeycrisp’ (**A**) and ‘Cripps Pink’ (**B**) from endodormancy induction to bud break stage. Red color indicates a positive correlation, whereas blue color indicates a negative correlation. The color intensity is proportional to the correlation coefficient.

ABA correlated significantly with trans-zeatin (−0.62, −0.69), starch (0.66, 0.57), NADPH oxidase (−0.55, −0.61), superoxide (−0.80, −0.71), and GSSG (−0.44, −0.73) in ‘Honeycrisp’ and ‘Cripps Pink’ respectively but no significant correlation was observed between H_2_O_2_ and ABA. ABA positively correlated with starch, but negatively correlated with the other molecules in both cultivars (Figure 6).

## 4. Discussion

Understanding the physiological mechanisms underlying apple bud dormancy is critical for developing strategies that can be used to modulate bloom time and thus avoid spring frost damage. In the present study, we used two apple cultivars, ‘Cripps Pink’ and ‘Honeycrisp’ with contrasting bloom dates to further our understanding of the biochemical pathways associated with bud dormancy and bloom time regulation in apple, aiming at providing a framework for devising agronomic strategies for modulating bud burst and bloom time.

### 4.1. Starch and Soluble Sugar Levels Differs between Apple Cultivars

Carbohydrates and their accumulation profiles in floral buds are tightly linked to the status of dormancy. Particularly, soluble sugars have been reported to act as signaling molecules [57] or physiological markers reflecting the depth of dormancy [26]. As a major carbon reserve form, starch content has been found to decrease with the progression of endodormancy concomitant with the increase of soluble sugars; presumably contributing to the increased cold hardiness in woody perennials [13,14,15,16,17,18]. Consistent with the above findings, our data showed an overall declining trend of starch content in both cultivars from the onset of endodormancy to budburst, with a marked peak at the release of endodormancy. Such increase in starch content upon the release of endodormancy was also found in the ovary of sweet cherry floral buds [58]. This synchronization of starch and chilling accumulation may also reflect the functional shift of starch, from being a carbohydrate reserve during endodormancy to a source of energy in the form of soluble sugars to promote active metabolism needed for bud break. Poplar mutant which accumulates higher level of sucrose displays advanced bud break [59]. Along the same vein, higher levels of starch and TSS were found in the early-blooming cultivar, ‘Cripps Pink’, during endodormancy. Generally, hexoses, such as glucose and fructose are released through the degradation of sucrose by acid invertase to provide cells with essential carbon and energy, for bud break [60]. The decrease in the TSS during ecodormancy in the both the cultivars support the hypothesis that storage carbohydrate is converted to structural carbohydrates, facilitating the resumption of growth [61]. In addition, soluble sugars, especially sucrose and fructose could serve as signaling entities during ecodormancy that promote the activity of vacuolar invertases and budburst [62]. Consistent with this notion is the higher levels of fructose in ‘Crisp Pink’ during ecodormancy indicating that fructose may be the dominant signaling hexose that contribute to growth resumption and budburst.

### 4.2. Hydrogen Peroxide May Act as a Biological Marker for Dormancy Transition

Accumulating evidence has suggested that the bud redox status resulting from the balance between generation and scavenging of ROS plays a critical role in bud dormancy transition [63]. As an important form of ROS, H_2_O_2_ has been suggested as a key signaling molecule associated with dormancy release. The characteristics of H_2_O_2_ that make it a potential molecule marker for chilling accumulation and dormancy status include membrane permeability, relatively long-life span, and oxidization of the critical thiol groups in some redox-sensitive proteins [64], which can theoretically lead to critical functional changes in proteins and transcription factors associated with dormancy. In addition, H_2_O_2_ is implicated in the control of plasmodesmata conductivity [65], a membrane-lined channels that regulates dormancy through dictating intracellular communication and transport of various cellular molecules [63]. In this study, the H_2_O_2_ level in ‘Cripps Pink’ peaked markedly when chilling requirement was fulfilled, confirming its role as an inducer of endodormancy release. However, such peak of H_2_O_2_ was not as prominent in ‘Honeycrisp’, which maintained at relatively high level until 2 weeks before bloom. Such divergent H_2_O_2_ profile during ecodormancy may, at least partially, explains the differences in bloom dates between the two cultivars. Indeed, in grapevine floral buds, maintaining a high level of H_2_O_2_, by inhibiting H_2_O_2_ degradation, leads to delayed budbreak [66]. Together, our data support the hypothesis that removal of H_2_O_2_ from the buds through antioxidative mechanisms is required for growth resumption [66]. Nevertheless, how H_2_O_2_ differentially regulate the release of endodormancy and ecodormancy warrants further investigation.

Superoxide is generated ubiquitously in cells during aerobic metabolism. High levels of superoxide have been shown to enhance energy production and amino acid metabolism, which are both required for growth resumption [48,67]. Superoxide production is mainly catalyzed by NADPH oxidase [48,68], which was found to facilitate the superoxide production during dormancy release in Arabidopsis seeds and potato tubers [33,34]. Our data suggest that superoxide may be responsible for the early bloom of ‘Cripps Pink’, which displayed significant increase toward the budburst earlier than ‘Honeycrisp’. Such increase of superoxide could be attributed to NADPH oxidase, whose activity was in parallel with the profile of superoxide.

Antioxidants, both in non-enzymatic and enzymatic forms play a key role in cellular redox homeostasis. One of the important non-enzymatic antioxidants in plants is glutathione. Metabolic activities of H_2_O_2_ scavenging systems require glutathione (GSH) that exists in the cells of dormant apple buds [69]. H_2_O_2_ detoxification through glutathione cycle requires NADPH that is generated via the pentose phosphate pathway (PPP). In this metabolic system, GSH participates as a reducing agent catalyzed by glutathione peroxidase (GPx), which results in reduction of H_2_O_2_ and formation of GSSG. The GSSG can then be reduced to GSH by glutathione reductase (GR) at the expense of NADPH [48,70]. In addition to GPX, H_2_O_2_ can also be detoxicated by CAT. Interestingly, CAT activity was found to be inhibited by the treatment of HC, leading to the accumulation of H_2_O_2_. In this study, the profiles of H_2_O_2_ levels in both cultivars bear high resemblance to the CAT activity, and to a lesser extent to the GSH activity, especially during the ecodormancy, suggesting that H_2_O_2_ may induce an immediate detoxication response, mediated primarily by CAT. In addition, this response appeared to be transient, rather than retained, as the CAT activity in ‘Honeycrisp’ decreased about 2 weeks (between 1000 and 2000 CH) earlier than the H_2_O_2_ levels during ecodormancy. In contrast to CAT, the profile of GPX activities was in opposite to H_2_O_2_ levels during both endodormancy and ecodormancy in both cultivars, indicating that GPX may operate in an antagonistic way with CAT in regulating H_2_O_2_.

Superoxide dismutase (SOD) quenches ROS by catalyzing the dismutation of superoxide radicals to O_2_ and H_2_O_2_. In apple buds, the SOD activity has been reported to rise toward the onset of budbreak [39,71]. In this study, the SOD activities were highly similar between ‘Cripps Pink’ and ‘Honeycrisp’ during both endodormancy and ecodormancy, with a slight increase before budburst. The similarity of SOD suggests SOD may play a less important role during dormancy in these two cultivars, in comparison to other ROS scavenging components, such GSH and CAT.

### 4.3. Interplay of ROS, Carbohydrates, and Hormones

Dormancy is a complex process that involves several interactions among key metabolic components including, but not limited to ROS, carbohydrate, and hormones. In our previous study, we showed that plant hormones abscisic acid (ABA) and cytokinin (CK) are critical regulators of bud dormancy and bloom in apple [49]. Comparison of the patterns of these hormones and those of carbohydrate and ROS found in this study could, to some extent, reveal their interactions and combined effects in controlling dormancy. Early study in white birch (*Betula pubescens*) showed that the starch content in the buds declines in concomitance with ABA during ecodormancy [72]. The close association between ABA and starch accumulation during endodormancy was also observed in grapevine buds [73]. In this study, despite some marked variations during the endodormancy, the accumulation pattern of starch generally mirrored that of ABA throughout the dormancy. In both cultivars, the positive correlations (0.66 and 0.57), between starch and ABA levels in ‘Honeycrisp’ and ‘Cripps Pink’, respectively, was significant.

ROS and ABA are both essential mediators of stress responses, and are also critical regulators of bud dormancy. In this study, the gradual decline of H_2_O_2_ during the early stage of endodormancy corresponds to the increase of ABA in both cultivars [49]. Additionally, the increase of superoxide level and NADPH oxidase activity both occurred only after ABA has reduced to its minimum level during ecodormancy. Such negative correlation between ROS and ABA has been demonstrated in other plant species. For example, ROS and ABA were found to act antagonistically in controlling dormancy and germination of barley seeds, as ABA suppresses the activity of NADPH oxidases [35] and enhances the activity of CAT [74]. In addition, H_2_O_2_ reduces ABA content by enhancing ABA-8′-hydroxylase, an ABA catabolic enzyme [74]. Similarly, under drought stress, ABA induces the CAT expression in rice leaves, which in turn prevents H_2_O_2_ accumulation, protecting cells against oxidative damage [75]. However, it is also worth noting that in other systems/plant tissues such as in Arabidopsis guard cells, ABA treatment induces the expression of NADPH oxidases [76], which in turn promotes ABA accumulation by enhancing ROS production, creating a positive feedback loop that mediates stomatal closure [77].

It is well established that CK is involved in the initial steps triggering growth resumption in floral buds [78]. Our previous study indicated that the different profiles of CK during ecodormancy may contribute to the contrasting bloom times between ‘Honeycrisp’ and ‘Cripps Pink’ [49]. In this study, the dynamics of superoxide and NADPH oxidase are highly similar to that of CK; both showing significant increase toward bud bust, with the increase occurring earlier in the early-blooming cultivar. The positive correlation between CK and ROS has been revealed in several studies with Arabidopsis. For example, CK was found to induce accumulation of ROS in guard cells of Arabidopsis through ABA-independent manner [79]. An Arabidopsis mutation with enhanced biosynthesis of CK resulted in ROS overproduction through transcriptional alteration of genes related to ROS scavenging and production [80].

## 5. Conclusions

The present study showed that differences between ‘Honeycrisp’ and ‘Cripps Pink’ apple cultivars in bloom time were associated with changes in the ROS and, to a lesser extent, carbohydrate accumulation profiles during the dormancy period. The H_2_O_2_ accumulation patterns closely correlated with the dormancy stage and bloom time in both cultivars, peaking early in the early-blooming cultivar while remaining at high levels for a longer time in the late-blooming cultivar. The findings of the present study match almost precisely similar results in grape and Japanese pear where the peak of H_2_O_2_ during dormancy marks the achievement of chilling requirements or the transition to another phase; reinforcing a notion that H_2_O_2_ either as a signaling molecule and/or a metabolite could serve as a molecular marker for the intensity of dormancy in deciduous woody perennials. In the present study, we also found that redox balance during dormancy was achieved mainly by catalase and, to a lesser extent, by GSH. The differential levels of superoxide in both apple cultivars, especially during ecodormancy, add another line of evidence that bloom time regulation in apple could, at least partially, be modulated by disrupting the bud redox balance. This study also revealed that the carbohydrate profiles between the two apple cultivars- though differing sporadically- may not sufficiently account for the differences in their bloom dates. However, it should also be noted that other forms of carbohydrates, such as sorbitol, were not studied in the present study, which warrants further investigation. Overall, the present study emphasizes that ROS and the redox balance in apple buds may serve as mediators of dormancy and bloom phenology among apple cultivars.

## Data Availability

Data are included in the article.

## References

[1] Heide O.M., Prestrud A.K. (2005). Low temperature, but not photoperiod, controls growth cessation and dormancy induction and release in apple and pear. Tree Physiol..

[2] Lang G. (1987). Dormancy: A new universal terminology. HortScience.

[3] Drepper B., Gobin A., Remy S., van Orshoven J. (2020). Comparing apple and pear phenology and model performance: What seven decades of observations reveal. Agronomy.

[4] Liu J., Islam M.T., Sapkota S., Ravindran P., Kumar P.P., Artlip T.S., Sherif S.M. (2021). Ethylene-mediated modulation of bud phenology, cold hardiness, and hormone biosynthesis in peach (*Prunus persica*). Plants.

[5] Persico M.J., Smith D.E., Centinari M. (2021). Delaying Budbreak to Reduce Freeze Damage: Seasonal Vine Performance and Wine Composition in Two *Vitis vinifera* Cultivars. Am. J. Enol. Vitic..

[6] Liu J., Sherif S.M. (2019). Combating Spring Frost with Ethylene. Front. Plant Sci..

[7] Ionescu I.A., Møller B.L., Sánchez-Pérez R. (2017). Chemical control of flowering time. J. Exp. Bot..

[8] Takeuchi T., Matsushita M.C., Nishiyama S., Yamane H., Banno K., Tao R. (2018). RNA-sequencing analysis identifies genes associated with chilling-mediated endodormancy release in apple. J. Am. Soc. Hortic. Sci..

[9] Wang S.Y., Faust M. (1988). Changes of fatty acids and sterols in apple buds during bud break induced by a plant bioregulator, thidiazuron. Physiol. Plant..

[10] Yu D.J., Jun S.H., Park J., Kwon J.H., Lee H.J. (2020). Transcriptome analysis of genes involved in cold hardiness of peach tree (*Prunus persica*) shoots during cold acclimation and deacclimation. Genes.

[11] Anderson J.V., Horvath D.P., Chao W.S., Foley M.E., Lubzens E., Cerda J., Clark M. (2010). Bud Dormancy in Perennial Plants: A Mechanism for Survival. Dormancy and Resistance in Harsh Environments.

[12] Sauter J.J., Wisniewski M., Witt W. (1996). Interrelationships between ultrastructure, sugar levels, and frost hardiness of ray parenchyma cells during frost acclimation and deacclimation in poplar (*Populus x canadensis* Moench 〈robusta〉) wood. J. Plant Physiol..

[13] Horikoshi H.M., Sekozawa Y., Kobayashi M., Saito K., Kusano M., Sugaya S. (2018). Metabolomics analysis of ‘Housui’ Japanese pear flower buds during endodormancy reveals metabolic suppression by thermal fluctuation. Plant Physiol. Biochem..

[14] Ito A., Sakamoto D., Moriguchi T. (2012). Carbohydrate metabolism and its possible roles in endodormancy transition in Japanese pear. Sci. Hortic..

[15] Rady M.M., Seif El-Yazal M.A. (2013). Response of “ Anna” apple dormant buds and carbohydrate metabolism during floral bud break to onion extract. Sci. Hortic..

[16] Sivaci A. (2006). Seasonal changes of total carbohydrate contents in three varieties of apple (*Malus sylvestris* Miller) stem cuttings. Sci. Hortic..

[17] Charrier G., Poirier M., Bonhomme M., Lacointe A., Améglio T. (2013). Frost hardiness in walnut trees *(Juglans regia* L.): How to link physiology and modelling?. Tree Physiol..

[18] Gholizadeh J., Sadeghipour H.R., Abdolzadeh A., Hemmati K., Hassani D., Vahdati K. (2017). Redox rather than carbohydrate metabolism differentiates endodormant lateral buds in walnut cultivars with contrasting chilling requirements. Sci. Hortic..

[19] Hamman R.A., Dami I.E., Walsh T.M., Stushnoff C. (1996). Seasonal carbohydrate changes and cold hardiness of chardonnay and riesling grapevines. Am. J. Enol. Vitic..

[20] Elle D., Sauter J.J. (2000). Seasonal changes of activity of a starch granule bound endoamylase and of a starch phosphorylase in poplar wood (*Populus x canadensis* Moench 〈robusta〉) and their possible regulation by temperature and phytohormones. J. Plant Physiol..

[21] El-Yazal M.A.S., Rady M.M. (2013). Foliar-applied Dormex^TM^ or thiourea-enhanced proline and biogenic amine contents and hastened breaking bud dormancy in “Ain Shemer” apple trees. Trees-Struct. Funct..

[22] Zhang Z., Zhuo X., Zhao K., Zheng T., Han Y., Yuan C., Zhang Q. (2018). Transcriptome Profiles Reveal the Crucial Roles of Hormone and Sugar in the Bud Dormancy of *Prunus mume*. Sci. Rep..

[23] Ben Mohamed H., Vadel A.M., Geuns J.M.C., Khemira H. (2010). Biochemical changes in dormant grapevine shoot tissues in response to chilling: Possible role in dormancy release. Sci. Hortic..

[24] Takemura Y., Kuroki K., Jiang M., Matsumoto K., Tamura F. (2015). Identification of the expressed protein and the impact of change in ascorbate peroxidase activity related to endodormancy breaking in *Pyrus pyrifolia*. Plant Physiol. Biochem..

[25] Hernández J.A., Díaz-Vivancos P., Acosta-Motos J.R., Alburquerque N., Martínez D., Carrera E., García-Bruntón J., Barba-Espín G. (2021). Interplay among antioxidant system, hormone profile and carbohydrate metabolism during bud dormancy breaking in a high-chill peach variety. Antioxidants.

[26] Fernandez E., Cuneo I.F., Luedeling E., Alvarado L., Farias D., Saa S. (2019). Starch and hexoses concentrations as physiological markers in dormancy progression of sweet cherry twigs. Trees-Struct. Funct..

[27] Sachdev S., Ansari S.A., Ansari M.I., Fujita M. (2021). Abiotic Stress and Reactive Oxygen Species: Generation. Antioxidants.

[28] Jajic I., Sarna T., Strzalka K. (2015). Senescence, stress, and reactive oxygen species. Plants.

[29] Pérez F.J., Vergara R., Rubio S. (2008). H_2_O_2_ is involved in the dormancy-breaking effect of hydrogen cyanamide in grapevine buds. Plant Growth Regul..

[30] Kuroda H., Sugiura T., Ito D. (2002). Changes in hydrogen peroxide content in flower buds of Japanese pear (*Pyrus pyrifolia* Nakai) in relation to breaking of endodormancy. J. Jpn. Soc. Hortic. Sci..

[31] Khalil-Ur-Rehman M., Wang W., Dong Y., Faheem M., Xu Y., Gao Z., Shen Z.G., Tao J. (2019). Comparative transcriptomic and proteomic analysis to deeply investigate the role of hydrogen cyanamide in grape bud dormancy. Int. J. Mol. Sci..

[32] Pérez F.J., Lira W. (2005). Possible role of catalase in post-dormancy bud break in grapevines. J. Plant Physiol..

[33] Liu B., Zhao S., Tan F., Zhao H., Wang D.D., Si H., Chen Q. (2017). Changes in ROS production and antioxidant capacity during tuber sprouting in potato. Food Chem..

[34] Müller K., Carstens A.C., Linkies A., Torres M.A., Leubner-Metzger G. (2009). The NADPH-oxidase AtrbohB plays a role in Arabidopsis seed after-ripening. New Phytol..

[35] Ishibashi Y., Kasa S., Sakamoto M., Aoki N., Kai K., Yuasa T., Hanada A., Yamaguchi S., Iwaya-Inoue M. (2015). A role for Reactive oxygen species produced by NADPH oxidases in the embryo and aleurone cells in barley seed germination. PLoS ONE.

[36] Nir G., Shulman Y., Fanberstein L., Lavee S. (1986). Changes in the Activity of Catalase (EC 1.11.1.6) in Relation to the Dormancy of Grapevine (*Vitis vinifera* L.) Buds. Plant Physiol..

[37] Porcher A., Guérin V., Leduc N., Lebrec A., Lothier J., Vian A. (2021). Ascorbate–glutathione pathways mediated by cytokinin regulate H_2_O_2_ levels in light-controlled rose bud burst. Plant Physiol..

[38] Porcher A., Guérin V., Montrichard F., Lebrec A., Lothier J., Vian A. (2020). Ascorbate glutathione-dependent H_2_O_2_ scavenging is an important process in axillary bud outgrowth in rosebush. Ann. Bot..

[39] Wang S.Y., Jiao H.J., Faust M. (1991). Changes in the activities of catalase, peroxidase, and polyphenol oxidase in apple buds during bud break induced by thidiazuron. J. Plant Growth Regul..

[40] Wang S.Y., Faust M. (1994). Changes in the antioxidant system associated with budbreak in “Anna” apple (*Malus domestica* Borkh.) buds. J. Am. Soc. Hortic. Sci..

[41] Ophir R., Pang X., Halaly T., Venkateswari J., Lavee S., Galbraith D., Or E. (2009). Gene-expression profiling of grape bud response to two alternative dormancy-release stimuli expose possible links between impaired mitochondrial activity, hypoxia, ethylene-ABA interplay and cell enlargement. Plant Mol. Biol..

[42] Walton E.F., Wu R.M., Richardson A.C., Davy M., Hellens R.P., Thodey K., Janssen B.J., Gleave A.P., Rae G.M., Wood M. (2009). A rapid transcriptional activation is induced by the dormancy-breaking chemical hydrogen cyanamide in kiwifruit (*Actinidia deliciosa*) buds. J. Exp. Bot..

[43] Considine M.J., Foyer C.H. (2014). Redox regulation of plant development. Antioxid. Redox Signal..

[44] Liu Y., Ye N., Liu R., Chen M., Zhang J. (2010). H_2_O_2_ mediates the regulation of ABA catabolism and GA biosynthesis in Arabidopsis seed dormancy and germination. J. Exp. Bot..

[45] Khalil-Ur-Rehman M., Sun L., Li C.X., Faheem M., Wang W., Tao J.M. (2017). Comparative RNA-seq based transcriptomic analysis of bud dormancy in grape. BMC Plant Biol..

[46] Liang D., Huang X., Shen Y., Shen T., Zhang H., Lin L., Wang J., Deng Q., Lyu X., Xia H. (2019). Hydrogen cyanamide induces grape bud endodormancy release through carbohydrate metabolism and plant hormone signaling. BMC Genom..

[47] Ionescu I.A., López-Ortega G., Burow M., Bayo-Canha A., Junge A., Gericke O., Møller B.L., Sánchez-Pérez R. (2017). Transcriptome and metabolite changes during hydrogen cyanamide-induced floral bud break in sweet cherry. Front. Plant Sci..

[48] Considine M.J., Foyer C.H. (2021). Oxygen and reactive oxygen species-dependent regulation of plant growth and development. Plant Physiol..

[49] Sapkota S., Liu J., Islam M.T., Ravindran P., Kumar P.P., Sherif S.M. (2021). Contrasting bloom dates in two apple cultivars linked to differential levels of phytohormones and heat requirements during ecodormancy. Sci. Hortic..

[50] Xu H., Ediger D. (2021). Rootstocks with Different Vigor Influenced Scion—Water Relations and Stress Responses in AmbrosiaTM Apple Trees (*Malus Domestica* var. Ambrosia). Plants.

[51] Lordan J., Fazio G., Francescatto P., Robinson T. (2017). Effects of apple (*Malus × domestica*) rootstocks on scion performance and hormone concentration. Sci. Hortic..

[52] Cesaraccio C., Spano D., Snyder R.L., Duce P. (2004). Chilling and forcing model to predict bud-burst of crop and forest species. Agric. For. Meteorol..

[53] Gibson P.G., Reighard G.L. (2002). Chilling requirement and postrest heat accumulation in peach trees inoculated with peach latent mosaic viroid. J. Am. Soc. Hortic. Sci..

[54] Edwards E.J., Downie A.F., Clingeleffer P.R. (2011). A Simple Microplate Assay to Quantify Non-Structural Carbohydrates of Grapevine Tissue. Am. J. Enol. Vitic..

[55] Bradford M.M. (1976). A Rapid and Sensitive Method for the Quantitation of Microgram Quantities of Protein Utilizing the Principle of Protein-Dye Binding. Anal. Biochem..

[56] Kaundal A., Rojas C.M., Mysore K.S. (2012). Measurement of NADPH Oxidase Activity in Plants. Bio-Protocol.

[57] Welling A., Palva E.T. (2006). Molecular control of cold acclimation in trees. Physiol. Plant..

[58] Fadón E., Herrero M., Rodrigo J. (2018). Dormant flower buds actively accumulate starch over winter in sweet cherry. Front. Plant Sci..

[59] Park J.Y., Canam T., Kang K.Y., Unda F., Mansfield S.D. (2009). Sucrose phosphate synthase expression influences poplar phenology. Tree Physiol..

[60] Ben Mohamed H., Vadel A.M., Geuns J.M.C., Khemira H. (2012). Carbohydrate changes during dormancy release in Superior Seedless grapevine cuttings following hydrogen cyanamide treatment. Sci. Hortic..

[61] Charrier G., Lacointe A., Améglio T. (2018). Dynamic modeling of carbon metabolism during the dormant period accurately predicts the changes in frost hardiness in walnut trees *Juglans regia* L.. Front. Plant Sci..

[62] Rabot A., Henry C., Ben Baaziz K., Mortreau E., Azri W., Lothier J., Hamama L., Boummaza R., Leduc N., Pelleschi-Travier S. (2012). Insight into the role of sugars in bud burst under light in the rose. Plant Cell Physiol..

[63] Beauvieux R., Wenden B., Dirlewanger E. (2018). Bud Dormancy in Perennial Fruit Tree Species: A Pivotal Role for Oxidative Cues. Front. Plant Sci..

[64] Or E., Vilozny I., Eyal Y., Ogrodovitch A. (2000). The transduction of the signal for grape bud dormancy breaking induced by hydrogen cyanamide may involve the SNF-like protein kinase GDBRPK. Plant Mol. Biol..

[65] Rutschow H.L., Baskin T.I., Kramer E.M. (2011). Regulation of solute flux through plasmodesmata in the root meristem. Plant Physiol..

[66] Pérez F.J., Noriega X., Rubio S. (2021). Hydrogen peroxide increases during endodormancy and decreases during budbreak in grapevine (*Vitis vinifera* l.) buds. Antioxidants.

[67] Scandroglio F., Tórtora V., Radi R., Castro L. (2014). Metabolic control analysis of mitochondrial aconitase: Influence over respiration and mitochondrial superoxide and hydrogen peroxide production. Free Radic. Res..

[68] Gilroy S., Suzuki N., Miller G., Choi W.G., Toyota M., Devireddy A.R., Mittler R. (2014). A tidal wave of signals: Calcium and ROS at the forefront of rapid systemic signaling. Trends Plant Sci..

[69] Kuroda H., Sagisaka S., Chiba K. (1990). Seasonal Changes in Peroxide-scavenging Systems of Apple Trees in Relation to Cold Hardiness. J. Jpn. Soc. Hortic. Sci..

[70] Foyer C.H., Lopez-Delgado H., Dat J.F., Scott I.M. (1997). Hydrogen peroxide- and glutathione-associated mechanisms of acclimatory stress tolerance and signalling. Physiol. Plant..

[71] Abassi N.A., Kushad M.M., Endress A.G. (1998). Active oxygen-scavenging enzymes activities in developing apple flowers and fruits. Sci. Hortic..

[72] Rinne P., Tuominen H., Junttila O. (1994). Seasonal changes in bud dormancy in relation to bud morphology, water and starch content, and abscisic acid concentration in adult trees of *Betula pubescens*. Tree Physiol..

[73] Rubio S., Noriega X., Pérez F.J. (2019). ABA promotes starch synthesis and storage metabolism in dormant grapevine buds. J. Plant Physiol..

[74] Ishibashi Y., Aoki N., Kasa S., Sakamoto M., Kai K., Tomokiyo R., Watabe G., Yuasa T., Iwaya-Inoue M. (2017). The interrelationship between abscisic acid and reactive oxygen species plays a key role in barley seed dormancy and germination. Front. Plant Sci..

[75] Ye N., Zhu G., Liu Y., Li Y., Zhang J. (2011). ABA controls H_2_O_2_ accumulation through the induction of OsCATB in rice leaves under water stress. Plant Cell Physiol..

[76] Kwak J.M., Mori I.C., Pei Z.M., Leonhard N., Angel Torres M., Dangl J.L., Bloom R.E., Bodde S., Jones J.D.G., Schroeder J.I. (2003). NADPH oxidase AtrbohD and AtrbohF genes function in ROS-dependent ABA signaling in arabidopsis. EMBO J..

[77] Mittler R., Blumwald E. (2015). The roles of ROS and ABA in systemic acquired acclimation. Plant Cell.

[78] Roman H., Girault T., Barbier F., Péron T., Brouard N., Pencík A., Novák O., Vian A., Sakr S., Lothier J. (2016). Cytokinins are initial targets of light in the control of bud outgrowth. Plant Physiol..

[79] Arnaud D., Lee S., Takebayashi Y., Choi D., Choi J., Sakakibara H., Hwang I. (2017). Cytokinin-mediated regulation of reactive oxygen species homeostasis modulates stomatal immunity in arabidopsis. Plant Cell.

[80] Wang Y., Shen W., Chan Z., Wu Y. (2015). Endogenous cytokinin overproduction modulates ROS homeostasis and decreases salt stress resistance in *Arabidopsis thaliana*. Front. Plant Sci..

